# Supplementary material for: Femtosecond laser programmed artificial musculoskeletal systems
Source: Nat Commun. 2020 Sep 10;11:4536. doi: 10.1038/s41467-020-18117-0 (PMC7484797; doi:10.1038/s41467-020-18117-0)
Supplement: Supplementary file 1 — Supplementary Information [file 41467_2020_18117_MOESM1_ESM.pdf]

Supporting Information

Femtosecond Laser Programmed Artificial Musculoskeletal Systems

Zhuo-Chen Ma[1,2], Yong-Lai Zhang[1]*, Bing Han[2], Xin-Yu Hu[1], Chun-He Li[1], Qi-Dai Chen[1], and Hong-Bo Sun[1, 2]*

[1] State Key Laboratory of Integrated Optoelectronics, College of Electronic Science and Engineering, Jilin University, 2699 Qianjin Street, Changchun 130012, China

[2] State Key Laboratory of Precision Measurement Technology and Instruments, Department of Precision Instrument, Tsinghua University, Haidian district, Beijing 100084, China.

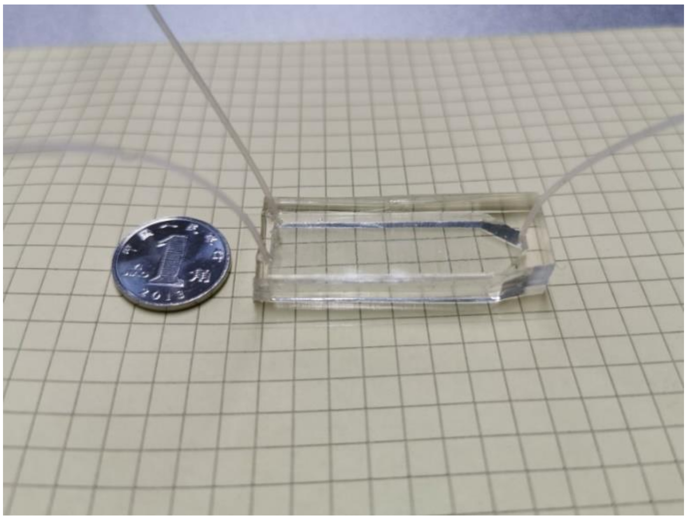

Supplementary Figure 1 | The photograph of the cover glass with a PDMS parapet for on-chip TPP. The parapet consists of a two inlet channels, for the injection of photoresist and developer, a PDMS chamber for TPP fabrication and developing, as well as one outlet channel for waste discharge.

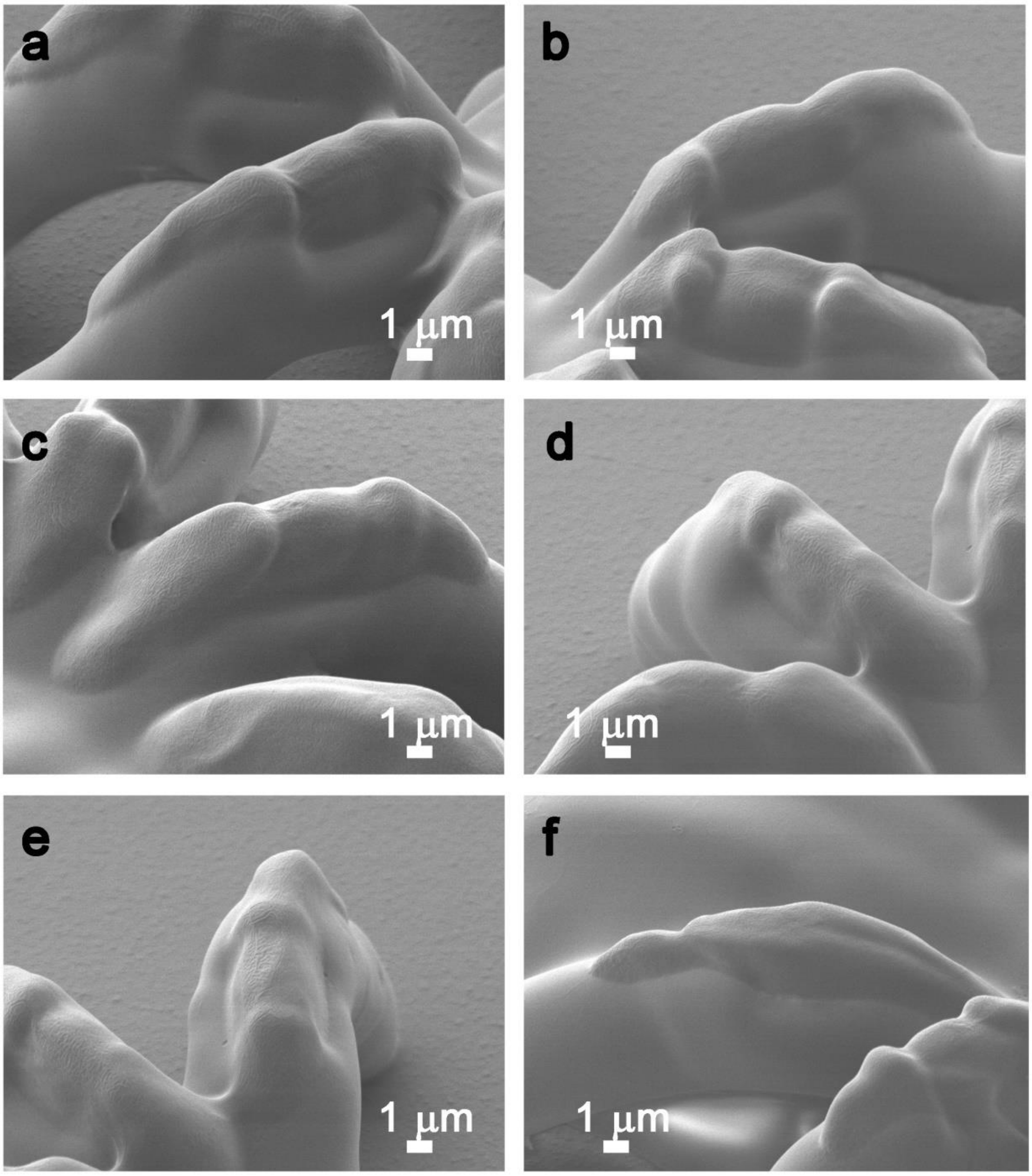

Supplementary Figure 2 | Detailed SEM images of the spider joints where the BSA muscles were integrated with the SU-8 legs. a-f, The joints on different legs of the micro-spider.

Experimental details for in-situ alignment of BSA muscles with SU-8 skeleton.

To realize in-situ integration of BSA muscles with SU-8 skeleton, the whole program of the musculoskeletal spider was divided into two complementary parts: skeleton and muscle. In the first run, a standard TPP process was carried out for the polymerization of the SU-8 spider skeleton with eight muscle vacancies. After that, the laser scanning program was suspended. Then in-situ development process was performed. As a developer, acetone was injected into the PDMS chamber to remove the unpolymerized photoresist. Subsequently, BSA gel was injected into the chamber for the second run of TPP fabrication. After that, the laser scanning program was restarted to continue with the laser scanning process of the BSA muscles. Since the program of the BSA muscles was well complementary to that of the SU-8 skeleton, re-positioning process is not necessary. The as-formed BSA voxels overlap well with the SU-8 skeleton at the vacancies. In this manner the muscles can be aligned accurately at the desired sites on the SU-8 spider.

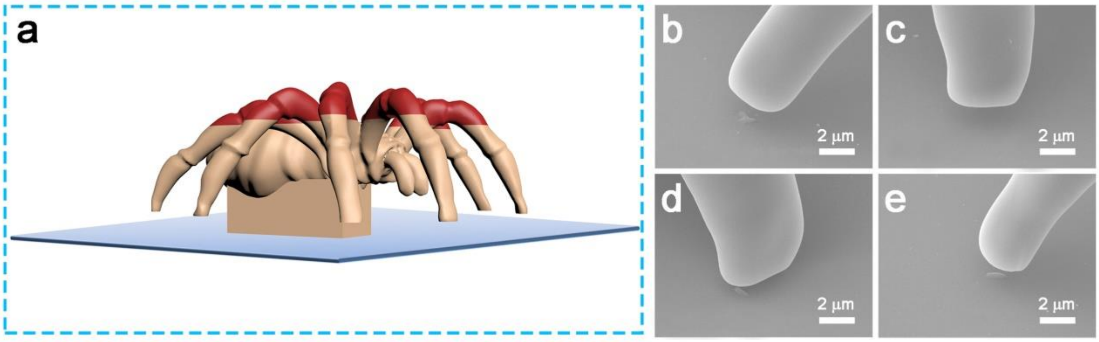

Supplementary Figure 3 | A 3D model of the spider microbot and the SEM images of the tip of the legs. a, Side view of the 3D model. **b-e**, Close-up SEM images of the tips of the legs.

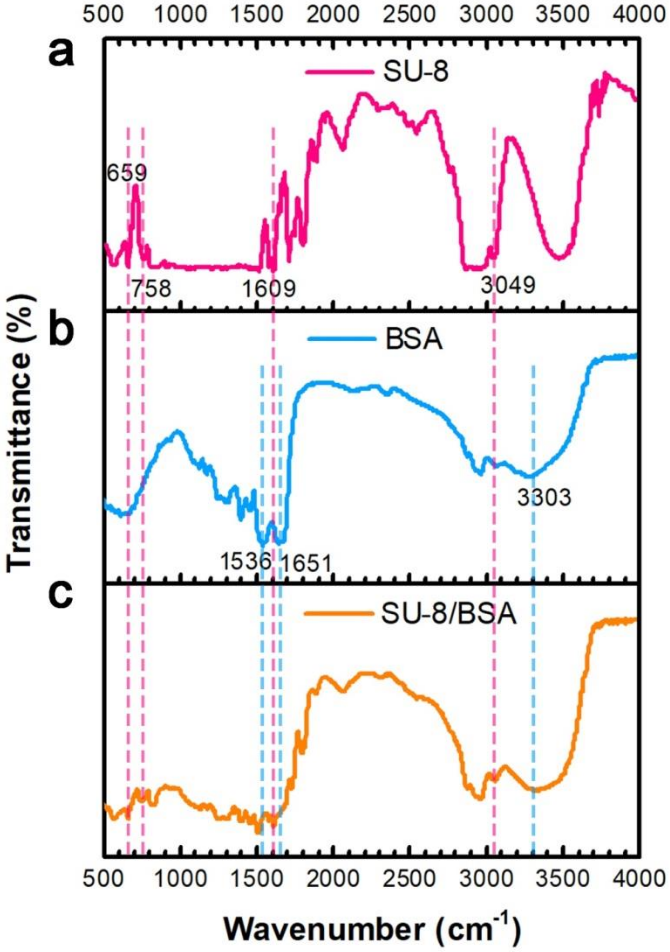

Supplementary Figure 4 | FTIR spectra of an SU-8 film (a), BSA film (b), and SU-8/BSA hybrid (c).

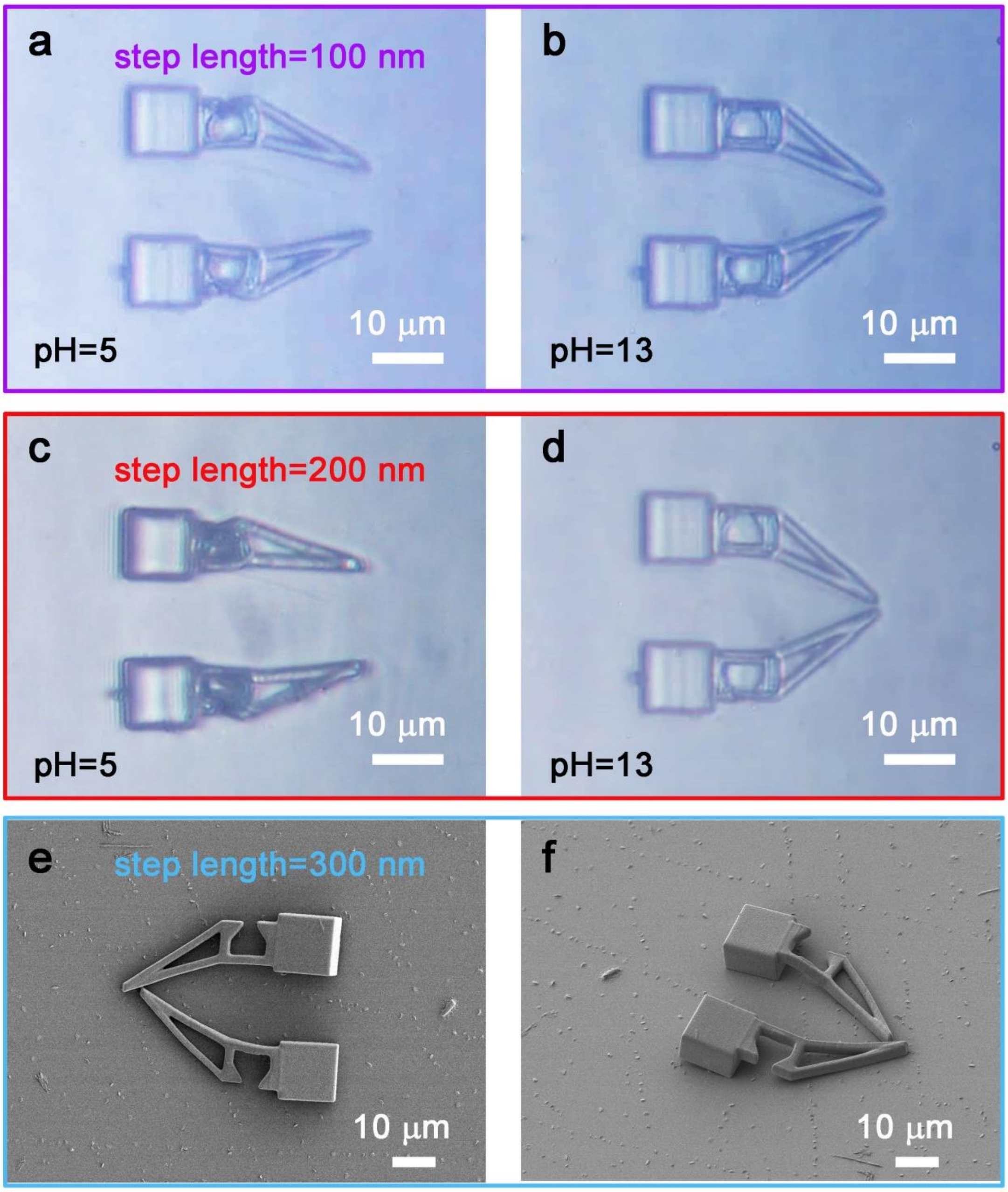

Supplementary Figure 5 | Comparison of the pH actuation of the micro-gripper with SU-8 skeletons fabricated using different laser scanning step lengths. a, b, The pH responsive performance of the micro-gripper with the SU-8 skeleton fabricated using a step length of 100 nm. **c, d** The pH responsive performance of the micro-gripper with the SU-8 skeleton fabricated using a step length of 200 nm. **e, f** SEM images of the SU-8 micro-gripper skeleton fabricated using a step length of 300 nm.

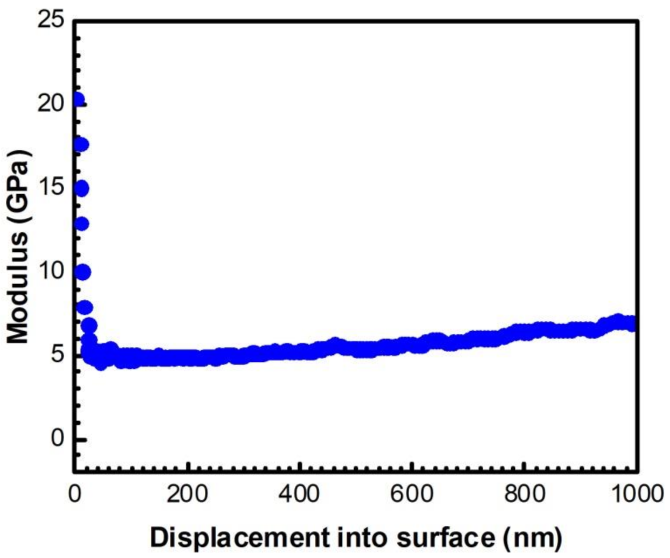

Supplementary Figure 6 | Young's modulus of SU-8 microstructures. The measurement was carried out at 30 ^{o}C with a relative humidity of 20%.

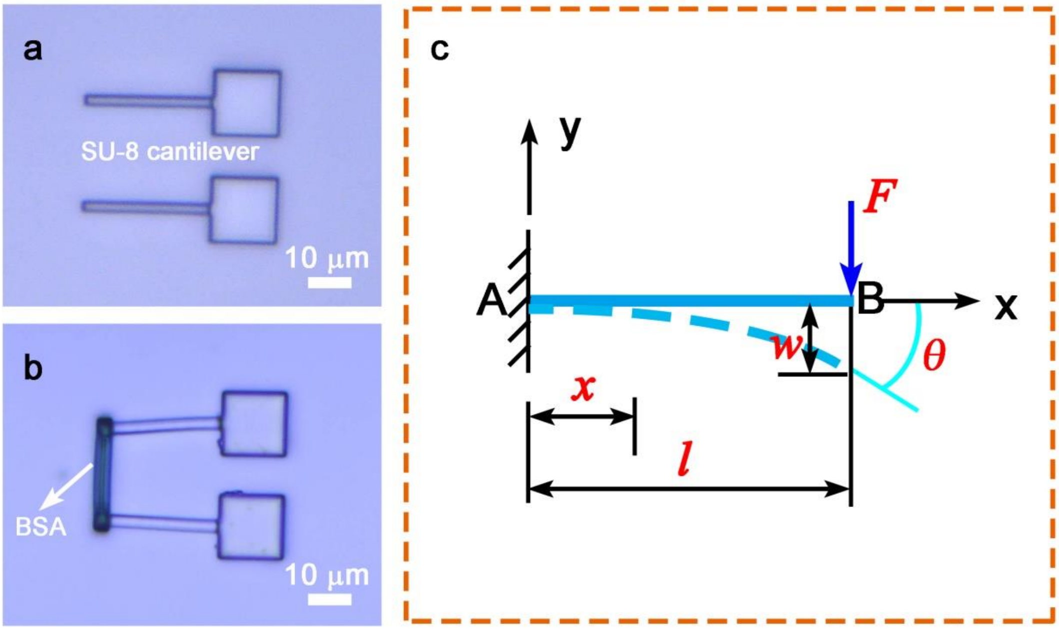

Supplementary Figure 7 | Experimental and mathematical analysis of the tension exerted by the BSA muscle on an SU-8 skeleton. a, Optical microscopy images of the SU-8 cantilevers. **b,** Optical microscopy images of the SU-8 cantilevers with a BSA muscle integrated at the free ends. **c,** The mathematical model based on approximate differential equation of the deflection curve.

Calculation of the tension exerted by the BSA muscle on an SU-8 cantilever:

The tension of the BSA muscle can be calculated according to the classical approximate differential equation of the deflection curve. The model is shown in Supplementary Fig. 3c, in which solid line *AB* represents the initial straight SU-8 cantilever without muscles (point *A* is the fixed end and point *B* is the free end), l represents the length of the cantilever, F represents the tension of the muscle exerted at the free end of the cantilever, dotted line represents the bended cantilever due to contraction or expansion of muscles, deflection w represents the linear displacement perpendicular to the x axis, and θ represents the rotation angle.

When the cantilever is bent, the relationship between curvature and bending moment is as follows:

$$\frac{1}{\rho} = \frac{M}{EI} \tag{1}$$

where ρ is the radius of curvature of the deformed cantilever, M is the bending moment acting on the cantilever, E is the modulus of elasticity of the cantilever material, and I is the modulus of inertia of the cross-sectional area about the neutral axis. M and ρ are both functions of x. Since the effect of shear force on the displacement of the cantilever can be omitted, then the following relationship exists,

$$\frac{1}{\rho(x)} = \frac{M(x)}{EI} \tag{2}$$

According to the geometric relationship, the curvature of a plane curve can be written as follows:

$$\frac{1}{\rho(x)} = \frac{w''}{\left(1+w'^2\right)^{3/2}} = \frac{M(x)}{EI} \tag{3}$$

Since the deflection curve is a very flat curve, w'^2 is much smaller than 1, which can be omitted, the above formula can be written as:

$$w'' = \frac{M(x)}{EI} \tag{4}$$

which is the approximate differential equation of the deflection curve. In our study the SU-8 cantilever is a straight beam with a constant cross section, hence the bending stiffness *EI* is a constant. Thus the above equation can be written as follows:

$$EIw'' = M(x) \tag{5}$$

The bending-moment equation of *x* section is as follows:

$$M(x) = -F(l-x) \tag{6}$$

Taking advantage of the approximate differential equation of the deflection curve, then the following equation exists:

$$EIw'' = -Fl + Fx \tag{7}$$

Then we can integrate the above formula once,

$$EI\frac{dw}{dx} = EI\theta = -Flx + \frac{Fx^2}{2} + C_1 \tag{8}$$

Next we can integrate the above formula again,

$$EIw = -\frac{Flx^2}{2} + \frac{Fx^3}{6} + C_1 x + C_2 \qquad (9)$$

The integral constant (C_1 and C_2) can be determined by using displacement boundary conditions:

$$\begin{cases} x = 0, w = 0 \\ x = 0, \theta = 0 \end{cases} \qquad (10)$$

then we can calculate the constant: C_1= 0, and C_2= 0.

Therefore, the deflection equation is as follows:

$$w = -\frac{Flx^2}{2EI} + \frac{Fx^3}{6EI} \qquad (11)$$

The deflection of point *B* at the free end is:

$$w_{x=l} = -\frac{Fl^3}{3EI} \qquad (12)$$

here l=30 μm, *E* is the modulus of elasticity of the cantilever material SU-8 (4.8 GPa), I=(2 μm)^4/12. If the tension *F*= 1.4 μN, then *w*= –1.96 μm; if *F*= 1.5 μN, *w*= –2.10 μm. In our study, the deflection *w* at the free end is ~ 2.05 ± 0.1 μm. Therefore, it can be calculated that the tension of the BSA muscle (with a width of 2 μm and a length of 25 μm) on the SU-8 cantilever is about 1.4~1.5 μN.

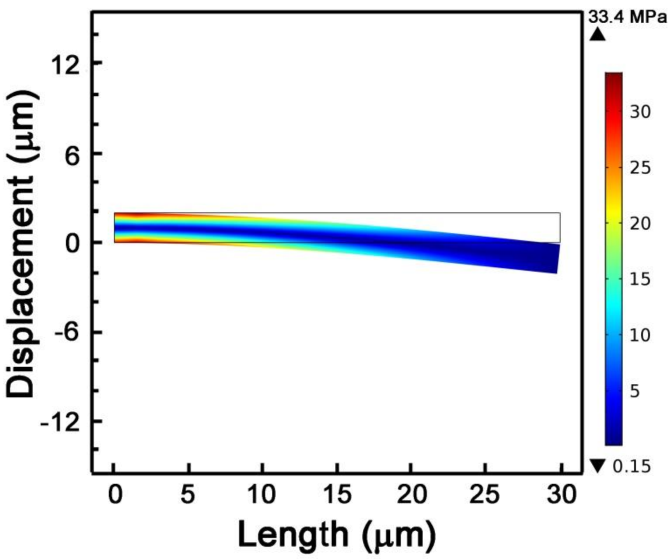

Supplementary Figure 8 | Simulation of the displacement of an SU-8 cantilever using COMSOL Multiphysics. The color bar represents the effective von Mises stress of the deformed cantilever beam with a force exerted at the free end on the right.

Simulation of the deflection of the cantilever beam:

In the model for simulation, the length of the SU-8 cantilever beam is 30 μm, and the cross section is a square with a side length of 2 μm. The cantilever is linear elastic with Young's modulus E= 4.8 GPa, Poisson's ratio ν=0.26, and density of 1200 kg m^{-3}. The left end is fixed, and the right end is subjected to a load of force F. This model is set up using both Solid Mechanics and Beam Interfaces. When the force F is set to be 1.4~1.5 μN, the tip displacement of the SU-8 cantilever is ~1.98~2.12 μm, which is consistent with the above mathematical calculation.

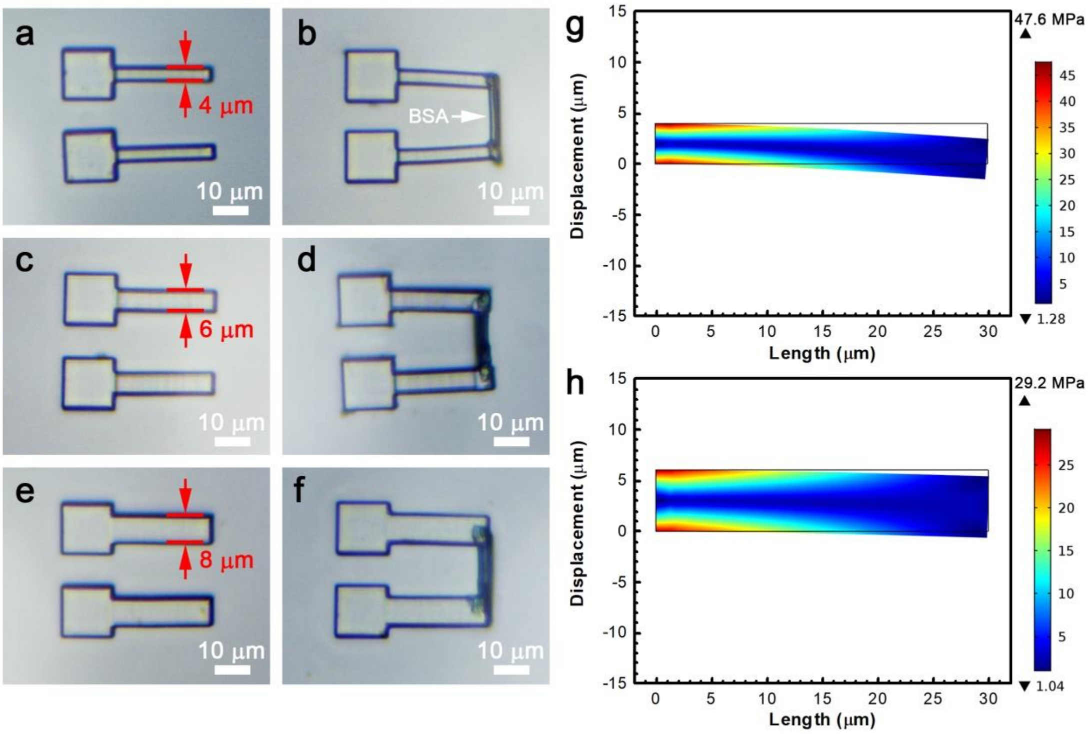

Supplementary Figure 9 | Experimental analysis and COMSOL simulation of the displacement of SU-8 cantilevers with different widths (4 μm, 6 μm, and 8 μm). a, b Optical microscopy images of the SU-8 cantilever with a width of 4 μm before and after integration of a BSA muscle at the free end. **c, d** Optical microscopy images of the SU-8 cantilever with a width of 6 μm before and after integration of a BSA muscle at the free end**. e, f** Optical microscopy images of the SU-8 cantilever with a width of 8 μm before and after integration of a BSA muscle at the free end**. g,** Simulation of the tip displacement of the SU-8 cantilever with a width of 4 μm when the exerted force on the tip is 17 μN. **h,** Simulation of the tip displacement of the SU-8 cantilever with a width of 6 μm when the exerted force on the tip is 34 μN.

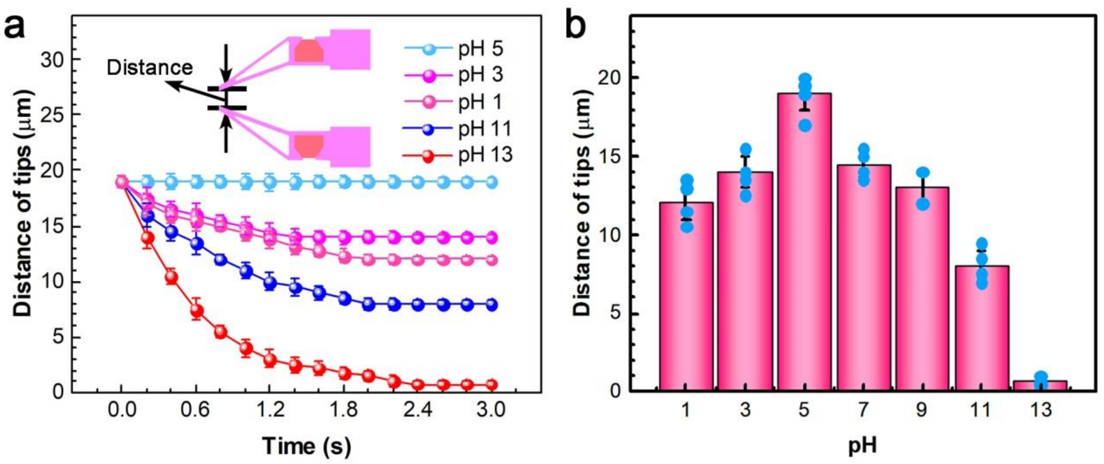

Supplementary Figure 10 | Dependence of the distance between two tips on pH value. a, Response time of the micro-gripper when the surrounding pH was changed from 5 to 1, 3, 11, and 13. **b,** Distance between two tips when the micro-gripper reaches steady state in solutions with different pH values (pH=1, 3, 5, 7, 9, 11, and 13). Error bars denote the standard deviation of the measurements.

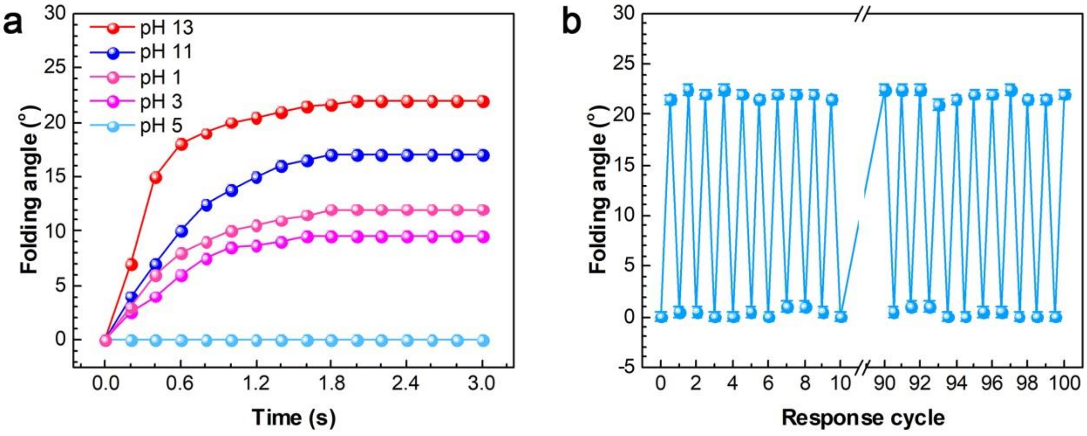

Supplementary Figure 11 | Dependence of the folding angle of the micro-gripper on pH values and the reversible response performance. a, Dynamic tuning process of the folding angle when the surrounding pH value was changed from 5 to 1, 3, 11, and 13. **b,** Variation of the folding angle in 100 response cycles when the pH value was switched reversibly from 13 to 5. Error bars denote the standard deviation of the measurements.

Supplementary Table 1 | The state-of-the-art of the reported micro-grippers.

Fabrication techniques	Materials	Size of devices	Actuation mechanism	Response time	Gripping capability	Ref.
**successive on-chip TPP strategy**	**SU-8/BSA**	**40 μm×35 μm**	**pH**	**~1 s**	**Pick and place micro-object (0~20 μm)**	**This work**
Dual-3D femtosecond laser nanofabrication	PBMA	D=40 μm	Acetone/ hexane	~1.5 s	Pick and place microsphere of ~ 20 μm	1
photolithography	PCL/ PNIPAM bilayers[a]	D=450 μm	Temperature	10 s	encapsulate and release yeast cells	2
photolithography, electrodeposition, and etching	Cr/Au bilayer, SC1805 and SC1813	D= ~ 980 μm	Temperature	10 min	tissue excision and biopsy of the bile duct	3
photolithography	PPF/ pNIPAM-AAc[b]	D= ~1 mm	Temperature	11 min	grip onto tissue and elute a drug	4
photolithography	POEGMA[c]	D= ~6 mm in	temperature	10 min	four-state shape changes	5
EBL[d], electron-beam evaporation and lift-off	SiO_2 and Fe	250 μm in length and 170 μm in width	Magnetic field	-	grip a single cell	6
photolithography	Au/Ni, Cr/ Cu/ novolac resin)	D= 700 μm	acetic acid and hydrogen peroxide	30 s	Pick and place beads (D=200 μm)	7
Photolithography, electroplating, and etching	Cr/Cu bilayer	D= ~ 1.2 mm	O_2 and H_2	25 s	bidirectional gripping	8
photolithography	Gelatin and CMC[e]	D= ~1.1 mm	enzyme	-	grip and release beads (D=700 μm)	9
Two-photon polymerization	Liquid crystal	D= ~200 μm	Light	0.1 s	Catch microcubes (40 × 40 × 20 μm^3)	10
photolithography	cresol novolak resin and a Cr/Cu thin film	D= ~700 μm	Temperature, glucose, and trypsin	40 s	removal of cells from tissue	11
photolithography	PEGDA[f]/NIPAAM bilayer with	D= ~400 μm	Temperature, light and	-	release of microparticles	12

	magnetic alginate microbeads		magnetic field			
photolithography	PHEMA[g] and PEGDA with Fe_3O_4 NPs	D= ~2 mm	Magnetic field and pH	1.5 min	Trap and release beads (D=300 μm)	13
Photolithography and thermal evaporation	SU-8/gold layer	2~3 mm long and 300~400 μm wide	Electrothermal	-	Cell manipulation	14
SOI process[h]	silicon	2900× 2700 μm	Electrostatic	-	Micro-object manipulation	15
μEDM[i], PECVD[j], and etching	Shape memory alloy (Ti/Ni) and SiO_2	33 mm ×9 mm × 3 mm	Electrothermal	7.5~9 s	Micro-object manipulation	16
MEMS processes (LPCVD, sputtering, and ICP-RIE)	sol-gel multi-coated PZT ($Pb(Zr_{0.52}Ti_{0.48})O_3$) films	2 mm-long and 100 μm wide	Piezoelectric	-	grip metallic balls (D= 100 μm)	17
electro-discharge machining	superelastic alloy (NiTi)	length 15.5 mm, width 5.22 mm, thickness 0.5 mm	Electromagnetic	-	tissues manipulation (~300 μm)	18
Photolithography and replica molding	polyurethane, magnetic microparticles, and hollow glass microbeads	700 μm in length and 70 μm in width	Magnetic field	-	Pick and place microgels	19
Two-photon polymerization	IP-Dip photoresist	~100 μm in length and breadth	External force applied to the center of the gripper	-	grasp, move and release objects (50×50 ×70 μm^3)	20
Photolithography and e-beam evaporation	SiO/SiO_2 bilayer	D= ~ 70 μm	residual stress within the prestressed bilayer	-	capture of live mouse cells	21

D: diameter

NPs: nanoparticles

[a] PCL: polycaprolactone

PNIPAM: poly-(N-isopropylacrylamide)

[b] PPF: poly(propylene fumarate)

pNIPAM-AAc: poly(N-isopropylacrylamide-co-acrylic acid)

[c] POEGMA: poly[oligo (ethylene glycol) methyl ether methacrylate]

[d] EBL: electron-beam lithography

[e] CMC: carboxymethylcellulose

[f] PEGDA: poly (ethylene glycol) acrylate

[g] PHEMA: 2-hydroxyethyl methacrylate

[h] SOI: silicon on insulator

[i] μEDM: micro-electrical-discharge-machining

[j] PECVD: plasma-enhanced chemical vapor deposition

Supplementary References

1. Zhang YL, *et al.* Dual-3D Femtosecond Laser Nanofabrication Enables Dynamic Actuation. *ACS Nano* **13**, 4041-4048 (2019).
2. Stoychev G, Puretskiy N, Ionov L. Self-folding all-polymer thermoresponsive microcapsules. *Soft Matter* **7**, (2011).
3. Gultepe E, *et al.* Biopsy with thermally-responsive untethered microtools. *Adv Mater* **25**, 514-519 (2013).
4. Malachowski K, *et al.* Stimuli-responsive theragrippers for chemomechanical controlled release. *Angew Chem Int Ed* **53**, 8045-8049 (2014).
5. Kobayashi K, Oh SH, Yoon C, Gracias DH. Multitemperature Responsive Self-Folding Soft Biomimetic Structures. *Macromol Rapid Commun* **39**, (2018).
6. Ger TR, Huang HT, Chen WY, Lai MF. Magnetically-controllable zigzag structures as cell microgripper. *Lab Chip* **13**, 2364-2369 (2013).
7. Randhawa JS, Leong TG, Bassik N, Benson BR, Jochmans MT, Gracias DH. Pick-and-place using chemically actuated microgrippers. *J Am Chem Soc* **130**, 17238-17239 (2008).
8. Randhawa JS, Keung MD, Tyagi P, Gracias DH. Reversible actuation of microstructures by surface-chemical modification of thin-film bilayers. *Adv Mater* **22**, 407-410 (2010).
9. Bassik N, *et al.* Enzymatically triggered actuation of miniaturized tools. *J Am Chem*

Soc **132**, 16314-16317 (2010).

10. Martella D, Nocentini S, Nuzhdin D, Parmeggiani C, Wiersma DS. Photonic Microhand with Autonomous Action. *Adv Mater* **29**, (2017).
11. Leong TG, Randall CL, Benson BR, Bassik N, Stern GM, Gracias DH. Tetherless thermobiochemically actuated microgrippers. *Proc Natl Acad Sci U S A* **106**, 703-708 (2009).
12. Fusco S, *et al.* An integrated microrobotic platform for on-demand, targeted therapeutic interventions. *Adv Mater* **26**, 952-957 (2014).
13. Li H, Go G, Ko SY, Park J-O, Park S. Magnetic actuated pH-responsive hydrogel-based soft micro-robot for targeted drug delivery. *Smart Mater Struct* **25**, (2016).
14. Solano B, Wood D. Design and testing of a polymeric microgripper for cell manipulation. *Microelectron Eng* **84**, 1219-1222 (2007).
15. Chang H, *et al.* A rotary comb-actuated microgripper with a large displacement range. *Microsystem Technologies* **20**, 119-126 (2013).
16. AbuZaiter A, Nafea M, Mohamed Ali MS. Development of a shape-memory-alloy micromanipulator based on integrated bimorph microactuators. *Mechatronics* **38**, 16-28 (2016).
17. Jeon C-S, Park J-S, Lee S-Y, Moon C-W. Fabrication and characteristics of out-of-plane piezoelectric micro grippers using MEMS processes. *Thin Solid Films* **515**, 4901-4904 (2007).
18. Kim D-H, Lee MG, Kim B, Sun Y. A superelastic alloy microgripper with embedded electromagnetic actuators and piezoelectric force sensors: a numerical and experimental study. *Smart Mater Struct* **14**, 1265-1272 (2005).
19. Chung SE, Dong X, Sitti M. Three-dimensional heterogeneous assembly of coded microgels using an untethered mobile microgripper. *Lab Chip* **15**, 1667-1676 (2015).
20. Power M, Thompson AJ, Anastasova S, Yang GZ. A Monolithic Force-Sensitive 3D Microgripper Fabricated on the Tip of an Optical Fiber Using 2-Photon Polymerization. *Small* **14**, e1703964 (2018).
21. Malachowski K, Jamal M, Jin Q, Polat B, Morris CJ, Gracias DH. Self-folding single cell grippers. *Nano Lett* **14**, 4164-4170 (2014).
