# Supplementary material for: Femtosecond laser programmed artificial musculoskeletal systems
Source: Nat Commun. 2020 Sep 10;11:4536. doi: 10.1038/s41467-020-18117-0 (PMC7484797; doi:10.1038/s41467-020-18117-0)
Supplement: Supplementary file 3 — Description of Additional Supplementary Files [file 41467_2020_18117_MOESM3_ESM.pdf]

Description of Additional Supplementary Files

File name: Supplementary Movie 1
Description: Actuation of the spider microbot (the pH value was switched from 13 to 5). Initially, the eight legs were bended since the BSA muscles were expanded at pH 13. When the pH value was switched to 5, the BSA muscles at the joints contracted, causing the straightening of the legs.

File name: Supplementary Movie 2
Description: Dynamic actuation performance of the arm-muscle system (the surrounding pH value was switched from 5 to 13). At the beginning, the arm was straight since the BSA muscle was contracted (pH=5). The BSA muscle expanded once the pH value was switched to 13, which caused the folding of the arm.

File name: Supplementary Movie 3
Description: Dynamic actuation of the crab claw-muscle system. Initially, the crab claw was open since the BSA muscle was contracted when the pH value was 5. After the pH value was switched to 13, the BSA muscle expanded, leading to the closing of the claw.

File name: Supplementary Movie 4
Description: A 3D smart micro-gripper that consists of the SU-8 skeleton and BSA muscle. Initially, the gripper was open since the BSA muscle was contracted (pH=5). Then the BSA muscle expanded once the pH value was switched to 13, which caused the gripping motion of the gripper.

File name: Supplementary Movie 5
Description: Targeted capturing, transport, and releasing of a cargo using the smart micro-gripper. First, the micro-gripper moved to the location of the target (a micro-cube with a side length of 10 μm). Then the pH value of the surrounding medium was switched to 13, which leads to the expansion of BSA muscles and the gripping of the gripper. Subsequently, the cube was held and transported to the desired location. Finally, the pH value was switched to 5, and the gripper released the cube due to the contraction of the BSA muscles.
